# Burden of Diabetes as a Contributing Cause in Dementia Mortality Among Older Adults in the United States, 1999–2020

**DOI:** 10.1002/brb3.71616

**Published:** 2026-07-23

**Authors:** Aasim Ali, Ayisha maqsood, Aswad Mustafa, Zeeshan Hussain, Areeba Ashraf, Sameen Sarfaraz, Ahmad Butt, Muhammad Shamoon, Labeeba Abdul Ghafoor, Hammad Azam, Muhammad Abdullah, Aimen Anwaar Pannun, Muhammad Asad Shabbir, Armaghana Abdullah, Mukesh Kumar Sharma

**Affiliations:** ^1^ Neurology Department Allied Hospital Faisalabad Faisalabad Pakistan; ^2^ Internal Medicine Department Allied Hospital Faisalabad Faisalabad Pakistan; ^3^ Warwick Medical School University of Warwick Coventry UK; ^4^ Neurology Department Dhankuta District Hospital Dhankuta Nepal

**Keywords:** Alzheimer's disease, CDC WONDER, dementia, diabetes mellitus, health disparities, mortality

## Abstract

**Background:**

Diabetes is a well‐established risk factor for cognitive decline and dementia; however, the extent to which diabetes is documented as a contributing cause on death certificates among decedents with dementia remains poorly characterized. This study aimed to evaluate temporal trends and demographic disparities in diabetes as a contributing condition among dementia‐related deaths in older adults in the United States.

**Methods:**

We analyzed multiple‐cause‐of‐death data from the CDC WONDER database for adults aged ≥ 65 years with dementia listed as an underlying or contributing cause of death between 1999 and 2020. Diabetes was identified using ICD‐10 codes E10–E14. We calculated age‐adjusted mortality rates (AAMR), crude death rates (CDR), annual percentage changes (APC) using Joinpoint regression, and the proportion of dementia deaths with coexisting diabetes. Analyses were stratified by age, sex, race, and state.

**Results:**

Among 3,818,272 dementia‐related deaths, 234,793 (6.2%) had diabetes documented as a contributing condition. The AAMR for dementia with diabetes increased from 8.7 per 100,000 in 1999 to 36.1 per 100,000 in 2020. Joinpoint regression identified a sharp increase from 1999 to 2009 (APC: +13.09%, 95% CI: 11.47–14.94, *p* < 0.001), followed by a stable trend from 2009 to 2020 (APC: +0.84%, 95% CI: −0.55–2.11, *p* = 0.187). Both dementia alone and dementia with diabetes demonstrated a sharp spike in 2020. The proportion of dementia deaths with coexisting diabetes was highest among decedents aged 65–74 years (8.5%) and decreased with age (85+ years: 5.3%). By race, American Indian/Alaska Native decedents had the highest proportion (10.4%), followed by Asian/Pacific Islander (9.2%), Black/African American (9.0%), and White (5.8%). Substantial state‐level variation was observed across the United States.

**Conclusions:**

The burden of diabetes, documented as a contributing condition among dementia‐related deaths, increased substantially from 1999 to 2009 and remained relatively stable thereafter. Significant racial, age, and geographic disparities exist, with American Indian/Alaska Native decedents showing the highest proportion of coexisting diabetes. These findings highlight the burden of metabolic comorbidity among older adults with dementia and underscore the importance of continued surveillance and targeted public health strategies.

## Graphical abstract

Among 3.8 million dementia‐related deaths in U.S. adults aged ≥65 years during 19992020, diabetes was documented in 6.2%. Mortality increased sharply until 2009 and then stabilized, with substantial age, racial/ethnic, and geographic disparities, highlighting the need for targeted prevention and continued surveillance.

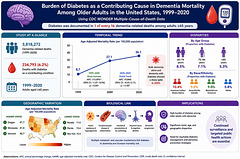

## 1 Introduction

The global burden of dementia is rising at an unprecedented pace, with an estimated 55 million people living with dementia worldwide, a number projected to triple by 2050 (GBD 2019 Dementia Forecasting Collaborators 2022; Alzheimer's Association 2023). In the United States, Alzheimer's disease and related dementias affect approximately 6.5 million older adults, making dementia one of the leading causes of death among individuals aged 65 years and older (Rajan et al. 2021; Kochanek et al. 2022). Concurrently, diabetes mellitus (DM) affects more than 37 million Americans, and its prevalence has been steadily increasing over the past two decades (Centers for Disease Control and Prevention 2022). Epidemiological studies have consistently demonstrated that diabetes is a significant risk factor for cognitive decline and dementia, with meta‐analyses showing a 50%–100% increased risk of Alzheimer's disease and vascular dementia among individuals with diabetes (Gudala et al. 2013; Chatterjee et al. 2016).

The pathophysiological link between diabetes and dementia is multifactorial, involving insulin resistance, chronic hyperglycemia, advanced glycation end‐products (AGEs), neuroinflammation, and cerebrovascular disease (Arnold et al. 2018; Kellar and Craft 2020). Diabetes contributes to both Alzheimer's disease pathology (via impaired amyloid‐β clearance and tau hyperphosphorylation) and vascular dementia (via small vessel disease, white matter lesions, and cerebral microinfarcts) (Biessels and Despa 2018; van Sloten et al. 2020). In addition, oxidative stress, mitochondrial dysfunction, blood‐brain barrier disruption, and impaired neuronal insulin signaling have been implicated in synaptic dysfunction and progressive cognitive decline, further supporting the close relationship between metabolic and neurodegenerative disorders (Arnold et al. 2018; Kellar and Craft 2020; Biessels and Despa 2018; van Sloten et al. 2020). Despite this well‐established biological connection, the extent to which diabetes is documented as a contributing cause on death certificates among decedents with dementia remains poorly characterized.

Previous studies using death certificate data have examined trends in dementia mortality (Li et al. 2021; Weuve et al. 2014) and diabetes mortality (Gregg et al. 2018) separately, but few have quantified the burden of diabetes as a contributing cause specifically among dementia‐related deaths. Understanding this intersection is critical for public health surveillance, resource allocation, and clinical management of comorbid conditions in aging populations. The Centers for Disease Control and Prevention (CDC) Wide‐Ranging Online Data for Epidemiologic Research (WONDER) database provides a unique opportunity to examine national trends in multiple‐cause‐of‐death data, allowing for the identification of both underlying and contributing causes of death (Centers for Disease Control and Prevention 2026). Although death certificate data cannot establish causal relationships or disease trajectories, they provide valuable population‐level insights into the coexistence and documentation of chronic conditions among older adults.

Therefore, the objectives of this study were to: (1) describe temporal trends in mortality among older adults with dementia and diabetes documented as a contributing condition from 1999 to 2020; (2) examine demographic disparities by age, sex, race, and geographic region; and (3) quantify the proportion of dementia‐related deaths with coexisting diabetes. We hypothesized that the burden of coexisting diabetes among dementia‐related deaths increased over time and that demographic disparities would exist across age, sex, race, and geographic regions.

## 2 Methods

### 2.1 Study Setting and Population

This study utilized mortality data from the CDC WONDER database (Chatterjee et al. 2016). We examined trends in mortality among individuals aged 65 years and older in whom dementia was recorded anywhere on the death certificate (as an underlying or contributing cause of death) and DM was documented as a contributing condition on US death certificates between 1999 and 2020. Data were extracted from the Multiple Cause of Death Public Use Files of the CDC WONDER database, a widely used resource for analyzing mortality patterns in which each death certificate includes one underlying cause of death and may contain multiple contributing conditions (Arnold et al. 2018).

Cases were identified using the International Statistical Classification of Diseases and Related Health Problems, 10th Revision (ICD‐10) codes. Dementia‐related deaths were defined using codes F01 (vascular dementia), F03 (unspecified dementia), and G30 (Alzheimer's disease). Diabetes documented as a contributing condition was identified using codes E10 (Type 1 DM), E11 (Type 2 DM), E13 (other specified DM), and E14 (unspecified DM). These classification codes have been employed in prior research investigating mortality linked to these conditions (Kellar and Craft 2020). Because ICD‐10 coding derived from death certificates does not permit assessment of disease chronology or causal relationships, the coexistence of dementia and diabetes in this study reflects documentation patterns rather than direct attribution of death to diabetes. Since the dataset is publicly available, de‐identified, and government‐provided, institutional review board approval was not necessary. This study was conducted following the Strengthening the Reporting of Observational Studies in Epidemiology (STROBE) guidelines (Biessels and Despa 2018).

### 2.2 Data Abstraction

This study categorized mortality data based on year, sex, age group (65–74, 75–84, and ≥ 85 years), race, and state of residence. Racial and ethnic groups were classified as American Indian or Alaska Native, Asian or Pacific Islander, Black or African American, and White, following the standard categorization used in previous CDC WONDER analyses and aligned with the US Office of Management and Budget guidelines (van Sloten et al. 2020). State‐level data included all 50 states and the District of Columbia.

### 2.3 Statistical Analysis

This study examined trends in mortality by calculating both crude death rates (CDR) and age‐adjusted mortality rates (AAMR) per 100,000 individuals along with their respective 95% confidence intervals (CI). AAMRs were standardized to the 2000 US standard population following established methodologies (Gregg et al. 2018). Given the descriptive nature of the study, emphasis was placed on point estimates and corresponding 95% CIs.

To evaluate temporal trends in mortality related to dementia with diabetes documented as a contributing condition, we utilized the Joinpoint Regression Program (Version 5.0.2, National Cancer Institute) (Centers for Disease Control and Prevention 2026). This method involved fitting log‐linear regression models to assess annual percentage change (APC) and its corresponding 95% CI. A trend was classified as increasing or decreasing if the slope significantly differed from zero, with statistical significance set at *p* ≤ 0.05 using a two‐tailed *t*‐test. Because CDC WONDER provides aggregate mortality data, information regarding glycemic control, diabetes duration, medication use, dementia severity, dementia subtype, and other clinical characteristics was unavailable.

## 3 Results

### 3.1 Overall Mortality Burden

Between 1999 and 2020, a total of 3,818,272 deaths among individuals aged 65 years and older had dementia recorded as the underlying or contributing cause of death. Among these, 234,793 deaths (6.2%) also had DM documented as a contributing condition. The overall CDR for dementia with coexisting diabetes was 25.3 per 100,000 population (95% CI: 25.2–25.4), and the overall AAMR was 25.4 per 100,000 population (95% CI: 25.3–25.5).

### 3.2 Temporal Trends

From 1999 to 2020, both the number of deaths and AAMR for dementia with diabetes documented as a contributing condition increased substantially. Deaths rose from 2995 in 1999 to 18,763 in 2020, representing a 6.3‐fold increase. The AAMR increased from 8.7 per 100,000 population (95% CI: 8.4–9.1) in 1999 to 36.1 per 100,000 population (95% CI: 35.6–36.7) in 2020.

**FIGURE 1:**
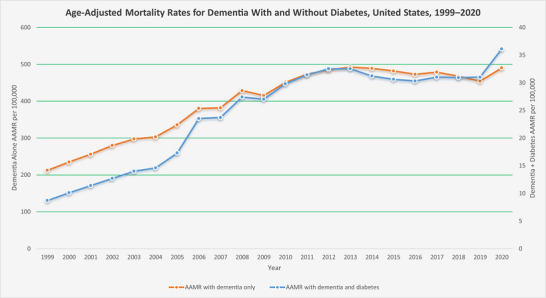
Temporal trends in age‐adjusted mortality rates (AAMR) for dementia with and without coexisting diabetes among adults aged ≥ 65 years, United States, 1999–2020. Dementia alone is shown on the left *Y*‐axis and dementia with coexisting diabetes on the right *Y*‐axis. Joinpoint analysis identified a change in 2009; AAMR increased from 1999 to 2009 (APC: +13.09%, 95% CI: 11.47–14.94; *p* < 0.001) and remained stable thereafter (APC: +0.84%, 95% CI: −0.55 to 2.11; *p* = 0.187). Both groups showed a marked increase in 2020.

Joinpoint regression analysis identified a significant change in trend in 2009. From 1999 to 2009, the AAMR for dementia with coexisting diabetes increased rapidly, with an APC of +13.09% (95% CI: 11.47–14.94; *p* < 0.001). From 2009 to 2020, the trend remained relatively stable, with an APC of +0.84% (95% CI: −0.55 to 2.11; *p* = 0.187). The AAMR for dementia alone increased from 1999 to 2013, declined from 2013 to 2019, and then increased sharply in 2020. These temporal trends are illustrated in Figure 1.

### 3.3 Age Distribution

The burden of dementia with coexisting diabetes increased markedly with advancing age. Decedents aged 85 years and older accounted for the majority of deaths (135,344 deaths; 57.6%), followed by those aged 75–84 years (81,135 deaths; 34.6%) and those aged 65–74 years (18,314 deaths; 7.8%). The crude mortality rate was highest among decedents aged 85 years and older (113.2 per 100,000 population; 95% CI: 112.6–113.8), followed by those aged 75–84 years (27.2 per 100,000 population; 95% CI: 27.0–27.4) and 65–74 years (3.6 per 100,000 population; 95% CI: 3.5–3.6).

**FIGURE 2:**
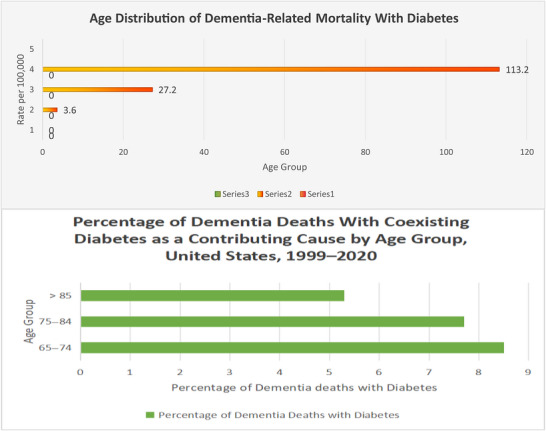
Age‐related patterns of dementia with coexisting diabetes among adults aged ≥ 65 years, United States, 1999–2020. (A) Crude mortality rates increased with age. (B) The proportion of dementia‐related deaths with coexisting diabetes was highest among adults aged 65–74 years (8.5%), followed by those aged 75–84 years (7.7%) and ≥ 85 years (5.3%).

When examining the proportion of dementia‐related deaths with coexisting diabetes, the youngest age group had the highest percentage. Diabetes was documented in 8.5% of dementia‐related deaths among decedents aged 65–74 years, followed by 7.7% among those aged 75–84 years and 5.3% among those aged 85 years and older. Age‐specific mortality rates and proportions are shown in Figure 2.

### 3.4 Sex Differences

Females accounted for a greater number of deaths from dementia with coexisting diabetes than males. Over the study period, 144,959 deaths occurred among females (61.7%) and 89,834 deaths occurred among males (38.3%). The crude mortality rate was higher among females (15.6 per 100,000 population) than males (9.7 per 100,000 population). However, AAMR were similar between males (25.4 per 100,000 population; 95% CI: 25.2–25.6) and females (25.3 per 100,000 population; 95% CI: 25.1–25.5).

**FIGURE 3:**
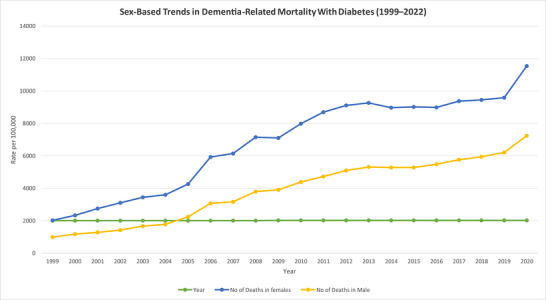
Sex‐specific trends in crude mortality rates for dementia with coexisting diabetes among adults aged ≥ 65 years, United States, 1999–2020. Mortality rates increased from 1999 to 2009 in both sexes and remained stable thereafter. Overall crude mortality rates were higher among females (15.6 per 100,000) than males (9.7 per 100,000).

The proportion of dementia‐related deaths with coexisting diabetes was slightly higher among females (6.3%) than males (5.9%). Temporal analysis by sex showed increasing crude mortality rates from 1999 to 2009, followed by relatively stable trends from 2009 to 2020 for both sexes. Sex‐specific temporal trends are shown in Figure 3.

### 3.5 Racial and Ethnic Disparities

Marked racial and ethnic differences were observed in mortality associated with dementia and coexisting diabetes. Black or African American decedents had the highest AAMR (35.5 per 100,000 population; 95% CI: 35.0–35.9), followed by White decedents (24.8 per 100,000 population; 95% CI: 24.7–24.9), American Indian or Alaska Native decedents (23.9 per 100,000 population; 95% CI: 22.5–25.3), and Asian or Pacific Islander decedents (18.5 per 100,000 population; 95% CI: 18.0–19.0).

By absolute number of deaths, White decedents accounted for the largest number of deaths (202,000 deaths; 86.0%), followed by Black or African American decedents (26,044 deaths; 11.1%), Asian or Pacific Islander decedents (5,624 deaths; 2.4%), and American Indian or Alaska Native decedents (1,125 deaths; 0.5%).

**FIGURE 4:**
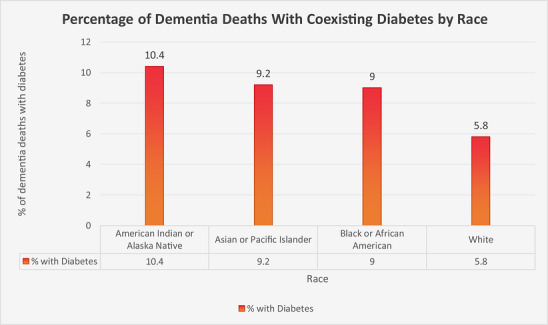
Percentage of dementia‐related deaths with coexisting diabetes by race, United States, 1999–2020. American Indian or Alaska Native decedents had the highest proportion (10.4%), followed by Asian or Pacific Islander (9.2%), Black or African American (9.0%), and White decedents (5.8%).

When examining the proportion of dementia‐related deaths with coexisting diabetes, American Indian or Alaska Native decedents had the highest percentage (10.4%), followed by Asian or Pacific Islander decedents (9.2%), Black or African American decedents (9.0%), and White decedents (5.8%). Racial and ethnic differences in the proportion of dementia‐related deaths with coexisting diabetes are presented in Figure 4.

### 3.6 Geographic Variation by State

Substantial geographic variation in AAMRs for dementia with coexisting diabetes was observed across the United States. The highest AAMRs were observed in Oregon (5164 deaths; AAMR: 41.7 per 100,000 population; 95% CI: 40.5–42.8), followed by Minnesota (6970 deaths; AAMR: 41.0 per 100,000 population; 95% CI: 40.1–42.0), Vermont (851 deaths; AAMR: 40.7 per 100,000 population; 95% CI: 37.9–43.4), Kentucky (4751 deaths; AAMR: 38.7 per 100,000 population; 95% CI: 37.6–39.8), and South Carolina (4843 deaths; AAMR: 37.7 per 100,000 population; 95% CI: 36.6–38.8).

**FIGURE 5:**
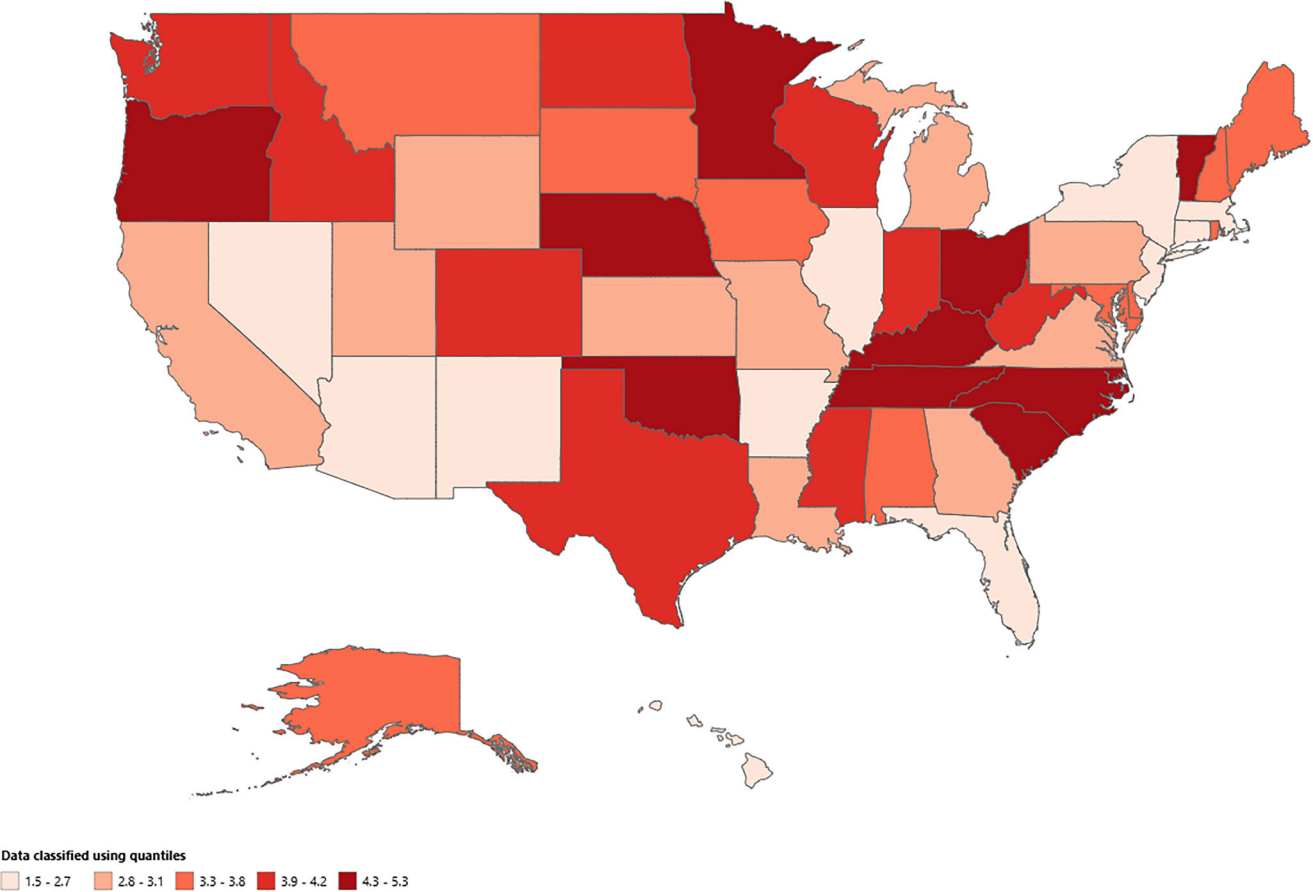
Geographic distribution of age‐adjusted mortality rates (AAMR) for dementia with coexisting diabetes by state, United States, 1999–2020. Highest AAMRs were observed in Oregon (41.7 per 100,000), Minnesota (41.0 per 100,000), and Vermont (40.7 per 100,000), whereas the lowest were observed in Nevada (11.5 per 100,000), Florida (13.6 per 100,000), and New York (15.4 per 100,000).

The lowest AAMRs were observed in Nevada (691 deaths; AAMR: 11.5 per 100,000 population; 95% CI: 10.6–12.4), Florida (10,449 deaths; AAMR: 13.6 per 100,000 population; 95% CI: 13.4–13.9), New York (9818 deaths; AAMR: 15.4 per 100,000 population; 95% CI: 15.1–15.7), Arizona (2977 deaths; AAMR: 15.7 per 100,000 population; 95% CI: 15.1–16.2), and New Jersey (4797 deaths; AAMR: 16.7 per 100,000 population; 95% CI: 16.2–17.1). Geographic variation in AAMR is shown in Figure 5.

## 4 Discussion

### 4.1 Principal Findings

In this national analysis of US multiple‐cause‐of‐death data from 1999 to 2020, we observed four principal findings. First, among more than 3.8 million dementia‐related deaths in adults aged ≥ 65 years, diabetes was documented as a coexisting contributing condition in approximately 6% of cases. Mortality rates for dementia with coexisting diabetes increased markedly between 1999 and 2009 and subsequently remained relatively stable through 2020. Second, both dementia alone and dementia with coexisting diabetes demonstrated a notable increase in 2020. Third, substantial disparities were observed across age, sex, race, and geographic regions, with the proportion of dementia‐related deaths with coexisting diabetes being highest among individuals aged 65–74 years and among American Indian or Alaska Native decedents. Finally, considerable state‐level variation in mortality rates was observed.

These findings highlight the burden of metabolic comorbidity among older adults with dementia and underscore the importance of continued surveillance and equitable public health strategies.

### 4.2 Biological Relationship Between Diabetes and Dementia

The association between diabetes and dementia is supported by several biological mechanisms and provides a plausible framework for understanding the coexistence of these conditions. Insulin resistance and chronic hyperglycemia contribute to oxidative stress, mitochondrial dysfunction, endothelial dysfunction, AGE accumulation, and chronic neuroinflammation, all of which may promote neuronal injury and cognitive decline. In addition, impaired insulin signaling has been implicated in abnormal amyloid‐β clearance and tau hyperphosphorylation, two pathological hallmarks of Alzheimer's disease (van Sloten et al. 2020; Li et al. 2021; Toniolo et al. 2021). Diabetes also contributes to cerebrovascular injury through accelerated atherosclerosis, microvascular disease, white matter lesions, and cerebral infarction, thereby increasing the risk of vascular cognitive impairment and vascular dementia (Toniolo et al. 2021; Power et al. 2018). These mechanisms provide biological plausibility for the close relationship between metabolic and neurodegenerative disorders and place our findings within a broader neuroscientific context.

However, because the present study relied on death certificate data, these biological pathways could not be directly evaluated, and the co‐documentation of diabetes and dementia should not be interpreted as evidence that diabetes directly caused death among individuals with dementia.

### 4.3 Temporal Trends and the Increase Observed in 2020

A major finding of this study was the marked increase in mortality rates from 1999 to 2009, followed by relatively stable trends thereafter. Although this pattern may reflect changes in disease prevalence, awareness, survival, or documentation practices, the underlying reasons cannot be determined from death certificate data alone. Therefore, causal explanations for the observed stabilization should be interpreted cautiously. Improvements in diabetes management and implementation of evidence‐based pharmacologic strategies over recent decades may have contributed to reduced diabetes‐related complications and mortality (American Diabetes Association 2021).

Another notable finding was the increase in both dementia‐related mortality and dementia with coexisting diabetes in 2020. This increase may partly reflect the multifactorial effects of the COVID‐19 pandemic, including excess mortality among vulnerable older adults, disruptions in healthcare access, delays in routine medical care, and possible changes in death certification practices (Stokes et al. 2021).

Because individual‐level information regarding SARS‐CoV‐2 infection, healthcare utilization, and disease trajectories was unavailable, the relative contribution of these factors cannot be determined. Continued surveillance will be important to establish whether the increase observed in 2020 represented a transient pandemic‐related phenomenon or part of a longer‐term trend.

### 4.4 Racial and Ethnic Disparities

One of the most important findings of our study was the marked racial and ethnic variation in the proportion of dementia‐related deaths with coexisting diabetes. American Indian or Alaska Native decedents exhibited the highest proportion, followed by Asian or Pacific Islander and Black or African American decedents.

Several factors may contribute to these disparities, including differences in diabetes prevalence, socioeconomic determinants of health, healthcare access, competing comorbidities, and variability in disease documentation (Cheng et al. 2019; Mayeda et al. 2016; Barnes and Bennett 2022; Wadhera et al. 2021). The elevated burden observed among American Indian or Alaska Native populations is particularly concerning given the longstanding health disparities experienced by these communities and the high prevalence of dementia‐related risk factors reported in these populations (Cline et al. 2021; Jernigan et al. 2020; Gone and Trimble 2012).

Our findings support the need for culturally appropriate prevention strategies and equitable access to diabetes management and cognitive health services. However, because death certificate data lack detailed clinical and socioeconomic information, the mechanisms underlying these disparities remain incompletely understood and warrant further investigation.

### 4.5 Age and Sex Patterns

Although most deaths occurred among individuals aged ≥ 85 years, the proportion of dementia‐related deaths with coexisting diabetes was highest among adults aged 65–74 years and declined with advancing age.

One possible explanation is that diabetes is more prevalent and more likely to be documented among younger elderly individuals, whereas older adults frequently experience substantial multimorbidity and competing causes of death that may reduce the likelihood that diabetes is recorded as a contributing condition. Previous studies have demonstrated that younger age at diabetes diagnosis and longer disease duration are associated with greater dementia risk (Zoungas et al. 2021). Age‐related differences in glycemic control may also contribute to these patterns (Selvin and Parrinello 2015).

Females accounted for a greater absolute number of deaths than males, largely reflecting the greater number of older women in the United States. However, AAMR were similar between sexes, suggesting that sex‐related differences were primarily attributable to demographic structure rather than differential disease burden.

The similarity in temporal trends between males and females suggests that factors influencing mortality patterns affected both sexes in a broadly comparable manner.

### 4.6 Geographic Variation

Substantial geographic variation in mortality rates associated with dementia and coexisting diabetes was observed across the United States. These differences may reflect variations in the prevalence of diabetes and dementia, socioeconomic characteristics, healthcare access, population demographics, and differences in documentation practices. Previous studies have similarly reported substantial regional variation in dementia mortality and differences in diagnosis and management patterns across healthcare systems (Cross et al. 2020; Gottlieb et al. 2021). Because completeness and accuracy of death certificate reporting may vary among states and population groups, geographic differences should be interpreted cautiously (McGivern et al. 2017). Future studies incorporating clinical registries and healthcare system characteristics may provide additional insight into the mechanisms underlying these regional patterns.

### 4.7 Comparison With Existing Literature

Our findings are broadly consistent with previous studies demonstrating increasing dementia‐related mortality in the United States. Prior investigations have examined dementia mortality and diabetes mortality separately; however, few studies have specifically evaluated diabetes documented as a coexisting contributing condition among dementia‐related deaths using multiple‐cause‐of‐death data. Thus, the present study extends previous work by characterizing long‐term temporal trends and demographic disparities in this population. Although mortality rates remained relatively stable after 2009, the underlying reasons cannot be determined from death certificate data. Consequently, these observations should be interpreted descriptively rather than as evidence of the effectiveness of specific interventions.

Observational studies have demonstrated that longer diabetes duration and poorer glycemic control are associated with accelerated cognitive decline and increased dementia risk (Zoungas et al. 2021; Power et al. 2018). In addition, metformin use has been associated with a lower incidence of dementia among older adults with diabetes (Samaras et al. 2021).

### 4.8 Clinical and Public Health Implications

Our findings underscore the importance of continued surveillance, early recognition of vascular risk factors, and equitable access to preventive and supportive healthcare services. Recommendations from the Lancet Commission emphasize risk‐factor modification and dementia prevention strategies across the life course (Livingston et al. 2020). Similarly, guidance for the care of older adults with multimorbidity highlights the need for individualized, patient‐centered approaches (American Geriatrics Society 2019). Development of dementia‐capable healthcare systems and efforts to reduce disparities in diagnosis and care have also been identified as public health priorities (Borson and Chodosh 2017; Chin et al. 2020). In addition, the observed racial and geographic disparities suggest that targeted public health interventions and culturally appropriate healthcare strategies may be necessary to reduce disparities among vulnerable populations (Borson and Chodosh 2017; Chin et al. 2020). Because death certificate data cannot establish causality, these findings should not be interpreted as evidence that diabetes directly caused death among individuals with dementia but rather as reflecting the burden of coexisting disease.

### 4.9 Strengths and Limitations

This study has several strengths. The use of the CDC WONDER database provided a nationally representative sample spanning 22 years and more than 3.8 million dementia‐related deaths, allowing robust analyses across age, sex, race, and geographic regions. The multiple‐cause‐of‐death approach captures both underlying and contributing conditions and provides a broader assessment of disease burden than analyses restricted to underlying causes alone. In addition, Joinpoint regression enabled characterization of temporal trends over time (Kim et al. 2000).

Several limitations should be acknowledged. First, death certificate data are susceptible to misclassification and underreporting. Dementia is frequently underreported on death certificates, and diabetes may not always be documented despite being present (Ives et al. 2009; McGivern et al. 2017). Therefore, the true burden of coexisting diabetes among individuals with dementia may be underestimated.

Second, because CDC WONDER provides cross‐sectional mortality data rather than longitudinal clinical information, disease trajectories and temporal relationships cannot be determined (Centers for Disease Control and Prevention 2026; Toniolo et al. 2021). Consequently, the coexistence of dementia and diabetes on death certificates should not be interpreted as evidence that diabetes directly caused death.

Third, age and multimorbidity represent important sources of confounding. Older adults frequently have multiple chronic conditions and competing causes of death, which may influence both disease documentation and mortality patterns (American Geriatrics Society 2019).

Fourth, the database lacks information regarding glycemic control, diabetes duration, HbA1c levels, medication use, atypical antipsychotic exposure, cognitive assessments, dementia severity, and other clinical characteristics (Toniolo et al. 2021, Power et al. 2018). These unavailable variables limit mechanistic interpretation.

Fifth, although ICD‐10 codes included several categories of diabetes, the present study could not reliably distinguish the effects of Type 1 and Type 2 diabetes, nor could we differentiate dementia severity or mixed pathological subtypes (Toniolo et al. 2021).

Finally, completeness and accuracy of death certificate reporting may vary among states and population groups, and misclassification of race and ethnicity on death certificates has been documented previously (Arias et al. 2016). These factors may influence regional and racial comparisons.

### 4.10 Future Directions

Future studies should focus on linking death certificate data with clinical registries and longitudinal cohorts to better characterize disease trajectories and causal relationships. Additional investigations are needed to clarify the mechanisms underlying racial and geographic disparities and to determine whether specific diabetes treatments, including metformin and newer glucose‐lowering therapies, influence dementia‐related outcomes (Samaras et al. 2021). Studies incorporating biomarkers, medication exposure, glycemic control, and measures of cognitive function may provide deeper insight into the relationship between metabolic dysfunction and neurodegeneration (Toniolo et al. 2021; Power et al. 2018). Continued surveillance will also be important to determine whether the increase observed in 2020 represented a transient pandemic‐related phenomenon or reflects longer‐term changes in mortality patterns.

### 4.11 Conclusions

In this national analysis of US death certificates from 1999 to 2020, the burden of diabetes as a contributing cause in dementia mortality increased sharply from 1999 to 2009, then plateaued from 2009 to 2020. In this national study of older adults with dementia, diabetes was frequently documented as a coexisting contributing condition. Mortality rates increased substantially from 1999 to 2009 and remained relatively stable thereafter, while marked age, racial, and geographic disparities were observed. These findings highlight the burden of metabolic comorbidity among older adults with dementia and emphasize the importance of continued surveillance and equitable public health strategies.

The reasons for the post‐2009 stabilization cannot be determined from death certificate data alone and require further investigation. Because death certificate data cannot establish causality, the observed coexistence of diabetes and dementia should be interpreted cautiously. Further longitudinal and mechanistic studies are needed to clarify the pathways linking metabolic dysfunction and neurodegeneration and to better understand disparities in this vulnerable population.

## Author Contributions

**Ayisha Maqsood**: conceptualization, writing – review and editing. **Ahmad Butt**: visualization, validation. **Labeeba Abdul Ghafoor**: writing – original draft, formal analysis. **Muhammad Shamoon**: investigation, project administration, data curation. **Muhammad Asad Shabbir**: funding acquisition, investigation. **Muhammad Abdullah**: methodology, software. **Areeba Ashraf**: formal analysis, methodology. **Sameen Sarfaraz**: writing – original draft, resources, data curation. **Aswad Mustafa**: investigation, formal analysis, supervision. **Hammad Azam**: formal analysis, investigation, supervision. **Zeeshan Hussain**: investigation, project administration. **Aimen Anwaar Pannun**: software, data curation. **Armaghana Abdullah**: resources, methodology, visualization, validation. **Mukesh Kumar Sharma**: writing – review and editing. **Aasim Ali**: conceptualization, methodology, writing – original draft.

## Funding

The authors have nothing to report.

## Ethics Statement

All summary‐level datasets used in our study were publicly available on CDC website; thus, no ethical approval was required.

## Conflicts of Interest

The authors declare no conflicts of interest.

## Data Availability

All data generated during this study is available on request. Contact the corresponding author if any information is required.

## References

[brb371616-bib-0001] Alzheimer's Association . 2023. “2023 Alzheimer's Disease Facts and Figures.” Alzheimer's & Dementia 19, no. 4: 1598–1695. 10.1002/alz.13016. 36918389

[brb371616-bib-0002] American Diabetes Association . 2021. “9. Pharmacologic Approaches to Glycemic Treatment: Standards of Medical Care in Diabetes—2021.” Supplement, Diabetes Care 44, no. S1: S111–S124. 33298420 10.2337/dc21-S009

[brb371616-bib-0003] American Geriatrics Society . 2019. “Guiding Principles for the Care of Older Adults With Multimorbidity.” Journal of the American Geriatrics Society 67, no. 10: 1995–2001.

[brb371616-bib-0004] Arias, E. , M. Heron , and J. K. Hakes , National Center for Health Statistics . US Census Bureau . 2016. “The Validity of Race and Hispanic‐Origin Reporting on Death Certificates in the United States: An Update.” Vital and Health Statistics 1, no. 172: 1–21. 28436642

[brb371616-bib-0005] Arnold, S. E. , Z. Arvanitakis , S. L. Macauley‐Rambach , et al. 2018. “Brain Insulin Resistance in Type 2 Diabetes and Alzheimer Disease: Concepts and Conundrums.” Nature Reviews Neurology 14, no. 3: 168–181. 10.1038/nrneurol.2017.185. 29377010 PMC6098968

[brb371616-bib-0006] Barnes, L. L. , and D. A. Bennett . 2022. “Racial and Ethnic Disparities in Dementia.” Continuum 28, no. 3: 862–878.

[brb371616-bib-0007] Biessels, G. J. , and F. Despa . 2018. “Cognitive Decline and Dementia in Diabetes Mellitus: Mechanisms and Clinical Implications.” Nature Reviews Endocrinology 14, no. 10: 591–604. 10.1038/s41574-018-0048-7. PMC6397437 30022099

[brb371616-bib-0008] Borson, S. , and J. Chodosh . 2017. “Developing Dementia‐Capable Health Care Systems: A 12‐Step Program.” Clinics in Geriatric Medicine 33, no. 3: 453–472. 10.1016/j.cger.2014.05.001 25037288

[brb371616-bib-0009] Centers for Disease Control and Prevention . National Diabetes Statistics Report 2022 . CDC. 2022.

[brb371616-bib-0010] Centers for Disease Control and Prevention . 2026. “CDC WONDER: About Multiple Cause of Death, 1999–2020.” Accessed April 3. https://wonder.cdc.gov/mcd‐icd10.html.

[brb371616-bib-0011] Chatterjee, S. , S. A. E. Peters , M. Woodward , et al. 2016. “Type 2 Diabetes as a Risk Factor for Dementia in Women Compared With Men: A Pooled Analysis of 2.3 Million People Comprising More Than 100,000 Cases of Dementia.” Diabetes Care 39, no. 2: 300–307. 10.2337/dc15-1588. 26681727 PMC4722942

[brb371616-bib-0012] Cheng, Y. J. , A. M. Kanaya , M. R. G. Araneta , et al. 2019. “Prevalence of Diabetes by Race and Ethnicity in the United States, 2011–2016.” JAMA 322, no. 24: 2389. 10.1001/jama.2019.19365. 31860047 PMC6990660

[brb371616-bib-0013] Chin, A. L. , S. Negash , and R. Hamilton . 2020. “Diversity and Disparity in Dementia Diagnosis and Care.” Neurologic Clinics 38, no. 4: 923–937.

[brb371616-bib-0014] Cline, M. J. , V. B. Jernigan , D. R. Babbage , et al. 2021. “Dementia Prevalence and Risk Factors Among American Indian and Alaska Native Populations: A Systematic Review.” Journal of Alzheimer's Disease 83, no. 4: 1531–1544.

[brb371616-bib-0015] Cross, S. H. , M. R. Mehra , and H. J. Warraich . 2020. “Geographic Variation in Dementia Mortality in the United States.” Journal of the American Geriatrics Society 68, no. 8: 1741–1748.

[brb371616-bib-0016] GBD 2019 Dementia Forecasting Collaborators . 2022. “Estimation of the Global Prevalence of Dementia in 2019 and Forecasted Prevalence in 2050: An Analysis for the Global Burden of Disease Study 2019.” Lancet Public Health 7, no. 2: e105–e125. 10.1016/S2468-2667(21)00249-8. 34998485 PMC8810394

[brb371616-bib-0017] Gone, J. P. , and J. E. Trimble . 2012. “American Indian and Alaska Native Mental Health: Diverse Perspectives on Enduring Disparities.” Annual Review of Clinical Psychology 8: 131–160. 10.1146/annurev-clinpsy-032511-143127. 22149479

[brb371616-bib-0018] Gottlieb, D. J. , A. Ellenbogen , M. Eckman , et al. 2021. “Regional Variation in the Diagnosis and Management of Dementia.” Journal of the American Geriatrics Society 69, no. 9: 2537–2546.

[brb371616-bib-0019] Gregg, E. W. , Y. J. Cheng , M. Srinivasan , et al. 2018. “Trends in Cause‐Specific Mortality Among Adults With and Without Diagnosed Diabetes in the USA: An Epidemiological Analysis of Linked National Survey and Vital Statistics Data.” Lancet 391, no. 10138: 2430–2440. 10.1016/S0140-6736(18)30314-3. 29784146

[brb371616-bib-0020] Gudala, K. , D. Bansal , F. Schifano , and A. Bhansali . 2013. “Diabetes Mellitus and Risk of Dementia: A Meta‐Analysis of Prospective Observational Studies.” Journal of Diabetes Investigation 4, no. 6: 640–650. 10.1111/jdi.12087. 24843720 PMC4020261

[brb371616-bib-0021] Ives, D. G. , P. Samuel , B. M. Psaty , and L. H. Kuller . 2009. “Agreement Between Nosologist and Cardiovascular Health Study Review of Deaths: Implications of Coding Differences.” Journal of the American Geriatrics Society 57, no. 1: 133–139. 10.1111/j.1532-5415.2008.02056.x. 19016930 PMC2631612

[brb371616-bib-0022] Jernigan, V. B. , E. J. D'Amico , B. Duran , and D. Buchwald . 2020. “Multilevel Approaches to Addressing Health Disparities in American Indian and Alaska Native Communities.” American Journal of Public Health 110, no. S1: S29–S30.

[brb371616-bib-0023] Kellar, D. , and S. Craft . 2020. “Brain Insulin Resistance in Alzheimer's Disease and Related Disorders: Mechanisms and Therapeutic Approaches.” Lancet Neurology 19, no. 9: 758–766. 10.1016/S1474-4422(20)30231-3. 32730766 PMC9661919

[brb371616-bib-0024] Kim, H. J. , M. P. Fay , E. J. Feuer , and D. N. Midthune . 2000. “Permutation Tests for Joinpoint Regression With Applications to Cancer Rates.” Statistics in Medicine 19, no. 3: 335–351. 10.1002/(SICI)1097-0258(20000215)19:3<335::AID-SIM336>3.0.CO;2-Z. 10649300

[brb371616-bib-0026] Kochanek, K. D. , S. L. Murphy , J. Q. Xu , and E. Arias . 2022. “Deaths: Final Data for 2020.” National Vital Statistics Reports 70, no. 9: 1–118. 37748091

[brb371616-bib-0027] Li, X. , J. Huang , Y. Zhang , et al. 2021. “Trends in Mortality From Alzheimer's Disease and Other Dementias in the United States, 1999–2019.” Journal of the Neurological Sciences 428: 117585.34371243

[brb371616-bib-0028] Livingston, G. , J. Huntley , A. Sommerlad , et al. 2020. “Dementia Prevention, Intervention, and Care: 2020 Report of the Lancet Commission.” Lancet 396, no. 10248: 413–446. 10.1016/S0140-6736(20)30367-6. 32738937 PMC7392084

[brb371616-bib-0029] Mayeda, E. R. , M. M. Glymour , C. P. Quesenberry , and R. A. Whitmer . 2016. “Inequalities in Dementia Incidence Between Six Racial and Ethnic Groups Over 14 Years.” Alzheimer's & Dementia 12, no. 3: 216–224. 10.1016/j.jalz.2015.12.007. PMC4969071 26874595

[brb371616-bib-0030] McGivern, L. , L. Shulman , J. K. Carney , S. Shapiro , and E. Bundock . 2017. “Death Certification Errors and the Effect on Mortality Statistics.” Public Health Reports 132, no. 6: 669–675. 10.1177/0033354917736514. 29091542 PMC5692167

[brb371616-bib-0031] Power, M. C. , A. Rawlings , A. R. Sharrett , et al. 2018. “Glycemic Control and Cognitive Decline in Older Adults With Diabetes.” JAMA Internal Medicine 178, no. 5: 662–670.

[brb371616-bib-0032] Rajan, K. B. , J. Weuve , L. L. Barnes , E. A. McAninch , R. S. Wilson , and D. A. Evans . 2021. “Population Estimate of People With Clinical Alzheimer's Disease and Mild Cognitive Impairment in the United States (2020–2060).” Alzheimer's & Dementia 17, no. 12: 1966–1975. 10.1002/alz.12362. PMC9013315 34043283

[brb371616-bib-0033] Samaras, K. , S. Makkar , J. D. Crawford , et al. 2021. “Metformin Use Is Associated With Reduced Risk of Dementia in Older Adults With Diabetes.” Diabetes Care 44, no. 12: 2739–2746.

[brb371616-bib-0034] Selvin, E. , and C. M. Parrinello . 2015. “Age‐Related Differences in Glycemic Control in Diabetes.” Current Diabetes Reports 15, no. 12: 105.26458376

[brb371616-bib-0035] Stokes, A. C. , D. J. Lundberg , K. Hempstead et al. 2021. “Trends in Cause of Death Certification in the United States, 1999–2019.” JAMA Network Open 4, no. 9: e2124771.

[brb371616-bib-0036] Toniolo, S. , L. Bello , F. Gallo , et al. 2021. “Diabetes and Dementia: A Critical Review of the Literature.” Journal of Clinical Medicine 10, no. 15: 3245.34362029

[brb371616-bib-0037] van Sloten, T. T. , S. Sedaghat , M. R. Carnethon , et al. 2020. “Cerebral Microvascular Complications of Type 2 Diabetes: Stroke, Cognitive Dysfunction, and Depression.” Lancet Diabetes & Endocrinology 8, no. 4: 325–336. 32135131 10.1016/S2213-8587(19)30405-X PMC11044807

[brb371616-bib-0038] Wadhera, R. K. , J. F. Figueroa , F. Rodriguez , et al. 2021. “Racial and Ethnic Disparities in Heart and Cerebrovascular Disease Mortality Among Medicare Beneficiaries.” JAMA Network Open 4, no. 9: e2124352.

[brb371616-bib-0039] Weuve, J. , L. E. Hebert , P. A. Scherr , and D. A. Evans . 2014. “Deaths in the United States Among Persons With Alzheimer's Disease (2010–2050).” Alzheimer's & Dementia 10, no. 2: e40–e46. 10.1016/j.jalz.2014.01.004. PMC3976898 24698031

[brb371616-bib-0040] Zoungas, S. , M. Woodward , Q. Li , et al. 2021. “Impact of Age, Age at Diagnosis, and Duration of Diabetes on the Risk of Dementia.” Diabetes Care 44, no. 7: 1589–1597.

